# Prions efficiently cross the intestinal barrier after oral administration: Study of the bioavailability, and cellular and tissue distribution *in vivo*

**DOI:** 10.1038/srep32338

**Published:** 2016-08-30

**Authors:** Akihiko Urayama, Luis Concha-Marambio, Uffaf Khan, Javiera Bravo-Alegria, Vineetkumar Kharat, Claudio Soto

**Affiliations:** 1 Mitchell Center for Alzheimer’s Disease and Related Brain Disorders, Department of Neurology, University of Texas Medical School at Houston, Texas, United States of America; 2 Universidad de los Andes, Facultad de Medicina, 2200 San Carlos de Apoquindo Av., Las Condes, Santiago 7620001, Chile

## Abstract

Natural forms of prion diseases frequently originate by oral (p.o.) infection. However, quantitative information on the gastro-intestinal (GI) absorption of prions (i.e. the bioavailability and subsequent biodistribution) is mostly unknown. The main goal of this study was to evaluate the fate of prions after oral administration, using highly purified radiolabeled PrP^Sc^. The results showed a bi-phasic reduction of PrP^Sc^ with time in the GI, except for the ileum and colon which showed sustained increases peaking at 3–6 hr, respectively. Plasma and whole blood ^125^I-PrP^Sc^ reached maximal levels by 30 min and 3 hr, respectively, and blood levels were constantly higher than plasma. Upon crossing the GI-tract ^125^I-PrP^Sc^ became associated to blood cells, suggesting that binding to cells decreased the biological clearance of the agent. Size-exclusion chromatography revealed that oligomeric ^125^I-PrP^Sc^ were transported from the intestinal tract, and protein misfolding cyclic amplification showed that PrP^Sc^ in organs and blood retained the typical prion self-replicating ability. Pharmacokinetic analysis found the oral bioavailability of ^125^I-PrP^Sc^ to be 33.6%. Interestingly, ^125^I-PrP^Sc^ reached the brain in a quantity equivalent to the minimum amount needed to initiate prion disease. Our findings provide a comprehensive and quantitative study of the fate of prions upon oral infection.

Prion diseases are a diverse group of fatal, infectious neurodegenerative disorders affecting both animals and humans^1^,^2^, including scrapie in sheep and goats, chronic wasting disease (CWD) in deer and elk, bovine spongiform encephalopathy (BSE) in cattle, and Creutzfeldt-Jakob disease (CJD) in humans. Scrapie is the most prevalent prion disease, first reported 200 years ago and endemic in several countries. The outbreak of BSE in the 1980s–90s led to the appearance of a new human disease, termed variant CJD (vCJD), which affected >200 people and peaked in 2000. Currently, it is still uncertain the number of people which remain as silent carriers of the infectious agent. Estimated prevalence of prion carriers in the UK from recent studies suggest a 1 in 2,000^3^. Concerns remain not only in the UK; indeed a recent study revealed continued necessity of global surveillance, based on the appearance of new vCJD cases in the US^4^. Another worrisome prion disease is CWD, a disorder affecting wild and captive cervids which continues to spread uncontrollably in North America. Very recently, the Norwegian Veterinary Institute reported the first case of CWD occurring in Europe. It is still unclear whether CWD prions can infect humans.

Prion diseases require long periods for the agent to replicate, reach the target organ and induce brain damage leading to the onset of clinical signs. The time between infection and clinical disease (termed incubation period) is variable and depend on the animal species, strain of the infectious agent, quantity of infectious material absorbed in the body, and route of administration. Tissue distribution of the infectious agent is largely confined to the CNS, although small amounts of the material can be consistently detected in peripheral tissues and biological fluids^2^.

Most of the cases of scrapie, CWD, BSE and vCJD arise from oral exposure through contaminated food and environmental materials. The oral route of transmission was demonstrated by infectivity studies with experimental animals deliberately exposed to prions by mouth^5^. After oral inoculation, the agent accumulates in the gut-associated lymphoid tissues such as the Peyer’s patches and mesenteric lymph nodes before neuroinvasion^5^,^6^,^7^,^8^,^9^. PrP^Sc^ is absorbed across the intestinal epithelium mediated through several mechanisms. Studies in Caco-2 cellular models of the intestinal barrier have shown that PrP^Sc^ can cross the barrier by endocytosis upon interaction with the laminin receptor^10^. On the other hand, Heppner and colleagues found that M cells mediate transcytosis of prions across the epithelial cell monolayers^11^. Mishra *et al*. showed that prion-ferritin complex is transcytosed in Caco-2 cell monolayers^12^. Kujala *et al*. found that the absorption of PrP^Sc^ through enteric lymphatic tissues was independent from constitutive expression of PrP^C ^13.

Regardless of the specific transcellular transport routes, once PrP^Sc^ crosses the intestinal barrier from the apical (mucosal) surface to the basolateral (serosal) side, PrP^Sc^ could be immediately absorbed by both dendritic cells and serousal fluid associated with mesenteric blood flow. Based on studies of infectivity and histological staining, it is generally assumed that the vasculature does not play a major role on the transport of PrP^Sc^ to the target organs, but instead that transmission involves direct uptake by peripheral nerves located in the oral or gastrointestinal mucosa^5^,^7^,^14^. However, previous experiments in which isolated gut loop were inoculated with prions, showed appearance of PrP^Sc^ rapidly in sub-mucosal lymphatics, much before detection in peyer’s patches of the intestine^15^. Since no evidence was obtained for the inoculum being transported across the dome to the peyer’s patches, the authors conclude that the most likely source of scrapie infection of lymphoid cells was blood. Moreover, since the pattern of PrP^Sc^ deposition does not change when the agent is administered by different routes and because early PrP^Sc^ accumulation was observed in circumventricular organs of sheep, it was proposed that the infectious agent may reach directly the brain from the blood through these structures where blood-brain barrier (BBB) is less tight^16^. Furthermore, a recent report from Denkers *et al*.^17^, showed that minor lesions in the oral cavity significantly facilitate CWD infection, suggesting that direct contact with blood circulation may play a role in prion spreading. In addition, the fact that prions acquired by oral exposure can be detected in various tissues, (including skeletal muscle) even before the onset of the disease^18^, is better explained by a hematogenous distribution of the infectious agent^19^. It is important to note that studies based on infectivity or conventional immuno-histochemical approaches do not have the sensitivity sufficient to elucidate the initial fate of PrP^Sc^ within minutes or hours after exposure; indeed, these methodologies usually rely on detection of prions after peripheral replication. Thus, no direct study has been done to assess how much of PrP^Sc^ given orally gets into the blood and what the fate is of ingested prions. In addition, it is unknown what percentage of PrP^Sc^ survives gut metabolism and is taken up from the intestinal lumen (i.e. the bioavailability of PrP^Sc^) and how the protein is then distributed in the body and reaches the brain.

Given the enormous social concerns, economic impact and public health consequences of prion transmission by infection and the unprecedented features of this protein-only infectious agent, it is highly surprising that this very important aspect of the disease pathogenesis has been almost completely neglected. Until now, the field has no quantitative information regarding the oral bioavailability, metabolism, and whole-body distribution profile of PrP^Sc^. The main goal of the present study was to analyze in a detailed and quantitative manner, the initial fate of prions upon oral exposure, including the estimation of oral bioavailability, spatio-temporal distributions throughout the GI tract, interaction with blood cells, uptake by peripheral organs and brain, thereby providing invaluable information to understand the pathogenesis of prion diseases which is important both for basic science and for risk assessment.

## Results

### Purity and size distribution of ^125^I-PrP^Sc^

To study the *in vivo* fate of PrP^Sc^ after oral administration, the protein was highly purified from the brain of mice infected with the RML prion strain, using a previously described procedure^20^. PrP^Sc^ purity was estimated to be >95% by silver staining and western blot (Fig. S1A). Thereafter, purified PrP^Sc^ was labeled with ^125^I, as previously described^20^. After radiolabeling, the molecular weight of ^125^I-PrP^Sc^ was analyzed by size-exclusion radio-HPLC chromatography (Fig. S1B). All the radioactivity measures after fractionation were normalized by TCA precipitation assay to separate free iodine and confirm that the peaks seen in the radio-chromatogram are protein associated. ^125^I-PrP^Sc^ appeared as one main speak with an estimated molecular weight of ~310 KDa and a minor peak of ~450 KDa (Fig. S1B), consisting with the expected size of the most infectious PrP^Sc^ species^21^. It is likely that larger fibrillar aggregates were removed during purification or labeling procedures. As previously reported, radiolabeling by iodination does not change PrP^Sc^ properties, including its resistance to proteolysis, biophysical stability against chemical denaturants and ability to self-propagate into infectious PrP^Sc^ material^20^. In addition, pharmacokinetic parameters of radiolabeled PrP^Sc^ were similar as those of full-length, unlabeled PrP^Sc ^20. As such, we could take advantage of the sensitivity of radiolabeled PrP^Sc^ to study the oral bioavailability and biodistribution in the present study.

### Gastro-intestinal distribution of PrP^Sc^

**Figure 1:**
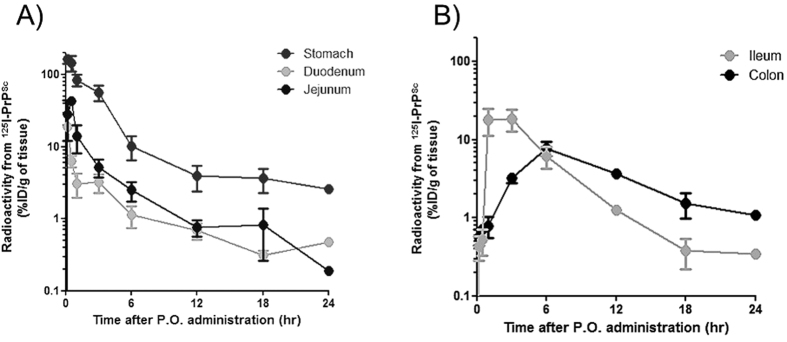
Time-courses of ^125^I-PrP^Sc^ level in the gastro-intestinal tract after p.o. administration. (**A**) Levels of ^125^I-PrP^Sc^ in the stomach, duodenum, and jejunum at different times after p.o. administration. (**B**) Levels of ^125^I-PrP^Sc^ in the ileum and colon, showing peak levels of ^125^I-PrP^Sc^ at 3 hr and 6 hr after p.o. administration, respectively. Experimentally naïve mice were fasted for 16–20 hr prior to the experiment. Mice received a p.o. administration of ^125^I-PrP^Sc^ (1 × 10^6^ cpm), and the whole blood, plasma, brain, liver, spleen, kidney, stomach, duodenum, jejunum, and colon was dissected. The radioactivity recovered from each tissue was further normalized by TCA precipitation to confirm the intactness of administered ^125^I-PrP^Sc^, and data expressed as %ID/g of tissue. Pearson’s correlation of tissue levels of ^125^I-PrP^Sc^ to the stomach levels over time were: duodenum (R^2^: 0.9684, ***P < 0.0001), jejunum (R^2^: 0.8940, **P = 0.0013), ileum (R^2^: 0.07014, P = 0.5660), and colon (R^2^:0.1916, P = 0.3259), respectively. Mean values with standard error terms are presented. (n = 4–7 per point). ID: Injected dose.

Experimentally naïve mice fasted for 16 to 20 hr received a p.o. administration of purified radiolabeled ^125^I-PrP^Sc^ using oral gavages. Intact levels of ^125^I-PrP^Sc^ were measured by normalizing radioactivity recovered from various tissues by TCA precipitation to negate overestimation from free radioactive iodine detached from ^125^I-PrP^Sc^. The results show a bi-phasic reduction in the levels of ^125^I-PrP^Sc^ in the stomach in fasted conditions (Fig. 1A). An initial phase of rapid reduction estimates the half-time of 1.29 hr followed by slower half-time of 25.9 hr. These findings suggest that, after relatively fast reduction of the majority of ^125^I-PrP^Sc^, remaining portion of PrP^Sc^ (around 3–4% of injected dose (ID)) was sequestered in the stomach and remained there even 24 hr after p.o. administration. The duodenum and jejunum showed similar patterns of ^125^I-PrP^Sc^ levels, which closely correlated with stomach over the study period (Fig. 1A). ^125^I-PrP^Sc^ in the duodenum showed a peak by 10 min after p.o. administration, and the levels decreased thereafter. The amount of ^125^I-PrP^Sc^ in this organ ranged from 18.7 to 0.3%ID/g. The jejunum showed similar retention of ^125^I-PrP^Sc^ to the duodenum, the maximal amount (43.4%ID/g) of ^125^I-PrP^Sc^ was found 30 min after the administration, thereafter the levels decreased with time (Fig. 1A). Interestingly, in the ileum where uptake of PrP^Sc^ (mediated through Payer’s patches and M cells) was reported to occur^11^, ^125^I-PrP^Sc^ was detected as fast as 10 min after p.o. administration, and the maximal levels (~18.4%ID/g) were reached by 1–3 hr followed by gradual decrease with time (Fig. 1B). The colon also showed small amount of ^125^I-PrP^Sc^ 10 min after administration, the levels peaked (~10%ID/g) at 6 hr, thereafter decreased to 2–3% ID/g by 24 hr. These findings suggest that fast distribution of ^125^I-PrP^Sc^ occurs in the GI-tract after oral ingestion, and that small amount of material are retained throughout the GI-tract over 24 hr.

### Whole blood retained higher levels of PrP^Sc^ mediated by blood cell association

**Figure 2:**
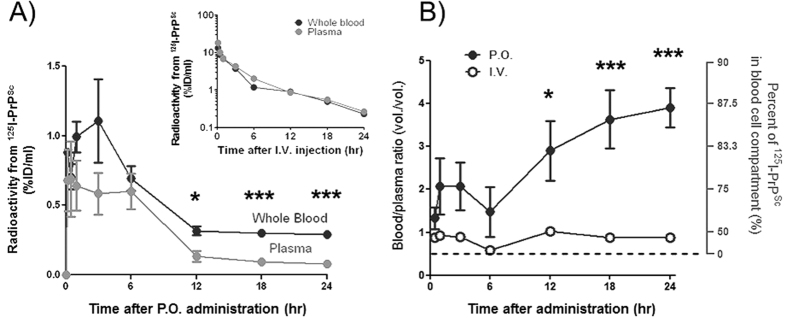
^125^I-PrP^Sc^ levels in whole blood and plasma after p.o. and i.v. administration. (**A**) Levels of PrP^Sc^ in whole blood and plasma after p.o. administration. Persistently higher levels of ^125^I-PrP^Sc^ in whole blood compared to plasma were observed after p.o. administration. The inset in panel A indicates the time-course of ^125^I-PrP^Sc^ levels after i.v. injection showing similar levels of ^125^I-PrP^Sc^ in the blood and plasma. (**B**) Blood-to-plasma ratios of ^125^I-PrP^Sc^ representing blood cell association of ^125^I-PrP^Sc^. While the ratios for ^125^I-PrP^Sc^ after the i.v. injection remained around 1, the ratios after p.o. administration increased with time. Percentages of cell-associated ^125^I-PrP^Sc^ after i.v. and p.o. administration were 14–48%, and 62–87%, respectively. Dashed line represents the ratio of 0.5, which corresponds to the ratio when all material remains in plasma. Blood collected at various time points during the same experiment described in Fig. 1 were separated in plasma and cellular package and radioactivity was quantified and corrected for TCA precipitation. Asterisks indicate statistical difference in the levels between the whole blood and plasma at various time points. Statistical difference was evaluated by two-tailed student T-test with Welch’s correction, and two-way ANOVA followed by the Bonferroni’s test. *P < 0.05, and ***P < 0.001. Mean values with standard error terms are presented. (n = 4–7). ID: Injected dose. Pharmacokinetic parameters for ^125^I-PrP^Sc^ after p.o. and i.v. administrations were calculated by these time-course profiles and summarized in Table 1.

We quantified intact ^125^I-PrP^Sc^ in the whole blood and plasma after p.o. or i.v. administrations, and blood cell associations of ^125^I-PrP^Sc^ observed by both routes were compared (Fig. 2). After i.v. injection, the levels of ^125^I-PrP^Sc^ in the whole blood were very similar as those seen in plasma and indeed the curves were not significantly different (Fig. 2A, inset). This result indicates that when PrP^Sc^ is directly deposited in blood, a high proportion of the material remains in the plasma. In contrast, the levels of ^125^I-PrP^Sc^ in the whole blood were constantly higher than that in plasma after p.o. administration (Fig. 2A), reaching statistical differences from 12 to 24 hr. Intact ^125^I-PrP^Sc^ in plasma peaked at 30 min (0.69%ID/ml) and the levels sustained by 6 hr, whereas in whole blood, intact ^125^I-PrP^Sc^ reached the maximal amount at 3 hr after p.o. administration.

**Figure 3:**
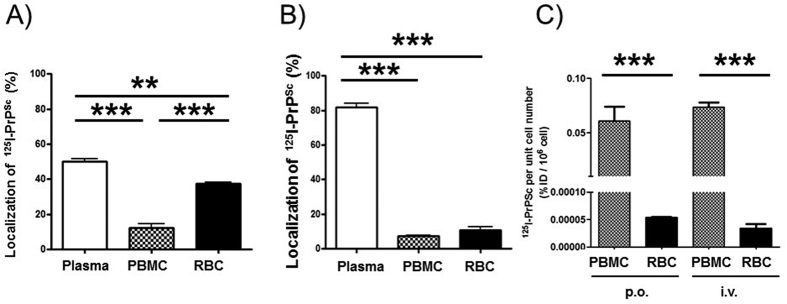
Percentage of ^125^I-PrP^Sc^ in various blood fractions after p.o. or i.v. administration. (**A**) Whole blood fractionation 3 hr after p.o. administration. (**B**) Whole blood fractionation 3 hr after i.v. injection. (**C**) The levels of ^125^I-PrP^Sc^ expressed by unit cell number in whole blood. ^125^I-PrP^Sc^ was administered at 1 × 10^6^ cpm in each route. Plasma, buffy coat (PBMC), and RBC fractions were freshly separated by Ficoll and TCA precipitable radioactivity was measured. Total radioactivity of intact ^125^I-PrP^Sc^ in whole blood was defined as 100%, and fractional percentage was calculated for each fraction. Statistical evaluation was performed with One-way ANOVA followed by Neuman-Keuls post-hoc test. **P < 0.01, and ***P < 0.001. Mean values with standard error terms are presented. (n = 3–4). ID: Injected dose.

The finding that the proportion of ^125^I-PrP^Sc^ in plasma and blood cells was different depending on the route of administration suggest that there is a large difference in blood cell association depending on the mode by which PrP^Sc^ get access into the blood. This is most clearly observed when blood/plasma ratios for intact ^125^I-PrP^Sc^ after i.v. and p.o. administrations were compared (Fig. 2B). While the ratios remained less than 1 for all time points in i.v. injected animals, indicating that only ~14–48% of ^125^I-PrP^Sc^ was cell associated, the ratios after p.o. administration increased with time, leading to an estimation that about 87% of ^125^I-PrP^Sc^ in blood got cell-associated at 24 hr (Fig. 2B). Two-way ANOVA revealed that these two administration routes produced statistically different time profiles of PrP^Sc^ in relation to cell association. At this point we cannot know whether ^125^I-PrP^Sc^ was taken up by certain type of blood cells or remained attached to the cell surface. Next, we further investigated the localization of ^125^I-PrP^Sc^ in whole blood by fractionating plasma, peripheral blood mononuclear cells (PBMC), and red blood cells (RBC) at the time (3 hr) when whole blood levels peaked after p.o. administration, and fractional distribution of ^125^I-PrP^Sc^ compared with i.v. injection (Fig. 3). In p.o. administration (Fig. 3A), about 44% of ^125^I-PrP^Sc^ existing in whole blood was distributed in the plasma fraction, 17% found in the PBMC, and 39% associated to RBC fraction, indicating that more than half (56%) of ^125^I-PrP^Sc^ in whole blood was eventually cell associated. In contrast, localization of ^125^I-PrP^Sc^ in whole blood after i.v. injection was markedly different; majority (80%) of ^125^I-PrP^Sc^ was found in plasma, and 7% and 13% was present in PBMC and RBC fractions, respectively (Fig. 3B). The explanation for the striking finding that the degree and manner of PrP^Sc^ cell association depends on the route of exposure, likely comes from differences in spatio-temporal absorption of ^125^I-PrP^Sc^ to blood. Figure 3C shows that the levels of ^125^I-PrP^Sc^ per respective cell counts in whole blood indicating about 1000-fold higher amount of the agent was accumulated in PBMCs compared to that of RBCs, regardless of the route of administration.

### Tissue distribution and oral bioavailability of PrP^Sc^

Pharmacokinetic parameters were calculated using a statistical moment analysis, which is pharmacokinetic model-independent approach, to estimate disposition parameters for ^125^I-PrP^Sc^, such as terminal half-life (t_1/2_, time in which 50% of ^125^I-PrP^Sc^ is eliminated from systemic circulation), steady state volume of distribution (Vdss, estimates the volume in which PrP^Sc^ distribute when the system has reached the equilibrium between tissue distribution and elimination), total body clearance rate (CLtot, estimates the rate in which PrP^Sc^ is removed from the body) and mean residual time (MRT represents the time required for clearing distributed ^125^I-PrP^Sc^ in the body) and oral bioavailability (Table 1). Considering that the levels of PrP^Sc^ in plasma and blood cells were different depending on the route of administration, we determined the pharmacokinetic parameters for both plasma and whole blood. While terminal half-lives of plasma ^125^I-PrP^Sc^ ranged 5.5 to 6.5 hr regardless of administration routes, prolonged terminal half-life (15.7 hr) was observed in whole blood after p.o. administration, indicating that cell association increased the terminal half-life of the agent. Mean residual time was estimated 20.7 hr based on whole blood profile in p.o. administration. This was mostly because of larger volumes of distribution at pharmacokinetic steady-state. The area under the curves for ^125^I-PrP^Sc^ by p.o. and i.v. routes were compared to estimate the percentage of ^125^I-PrP^Sc^ absorbed through the GI tract into the body, which is defined as absolute bioavailability of ^125^I-PrP^Sc^. Estimated bioavailability of ^125^I-PrP^Sc^ based on whole blood was 33.6% which was 2.4-fold higher than plasma based value of 14.3% (Table 1). The rate of the bioavailability was also shown as Tmax and Cmax values which refer to the time to reach maximal amount and the maximal concentration at Tmax, respectively.

**Figure 4:**
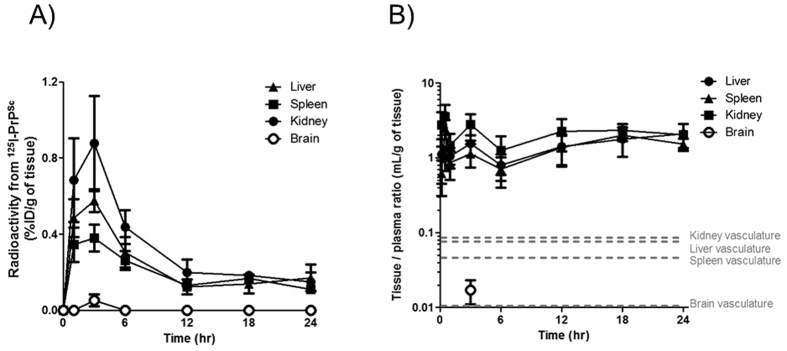
Brain and peripheral tissue distributions of ^125^I-PrP^Sc^ after p.o. administration. (**A**) Time-courses of TCA precipitable ^125^I-PrP^Sc^ in diverse tissues. Tissue levels showed similar time-profile seen in whole blood. Brain levels of ^125^I-PrP^Sc^ peaked at 3 h, and other time points were too low to be accurately measured. (**B**) The volumes of distribution of ^125^I-PrP^Sc^ represent the uptake of ^125^I-PrP^Sc^ over time with regard of tissue vascular space. Dashed lines indicate vascular space in each tissue estimated separately by i.v. injection of ^125^I-albumin. It is of note that tissue vascular space was similar to that obtained in our previous study 20. Each tissue persistently showed 9–10 times higher ^125^I-PrP^Sc^ distribution compared to its vascular space. Mean values with standard error are presented (n = 4). ID: injected dose.

Once ^125^I-PrP^Sc^ became blood-borne after p.o. administration, systemic circulation allows the delivery of prions throughout the body. We investigated the distributions of ^125^I-PrP^Sc^ in brain and peripheral organs (Fig. 4). The levels in the liver, spleen, and kidney showed a similar time profile as in systemic circulation (Fig. 2A). The volumes of ^125^I-PrP^Sc^ distribution were 9–10 times higher than the vascular space in each tissue measured separately by ^125^I-albumin (Fig. 4B), suggesting that the majority of PrP^Sc^ found in these tissues was indeed in the tissue and not in the blood associated to the tissue. The quantity of material reaching the brain was very low, but measurable at 3 hr after p.o. administration which is in agreement with prior studies showing that PrP^Sc^ can cross full-width of the blood-brain barrier^20^,^22^,^23^. Brain distributed ^125^I-PrP^Sc^ was ~1.5 fold higher than vascular space in the brain.

### Absorption of oligomeric PrP^Sc^ into blood stream

**Figure 5:**
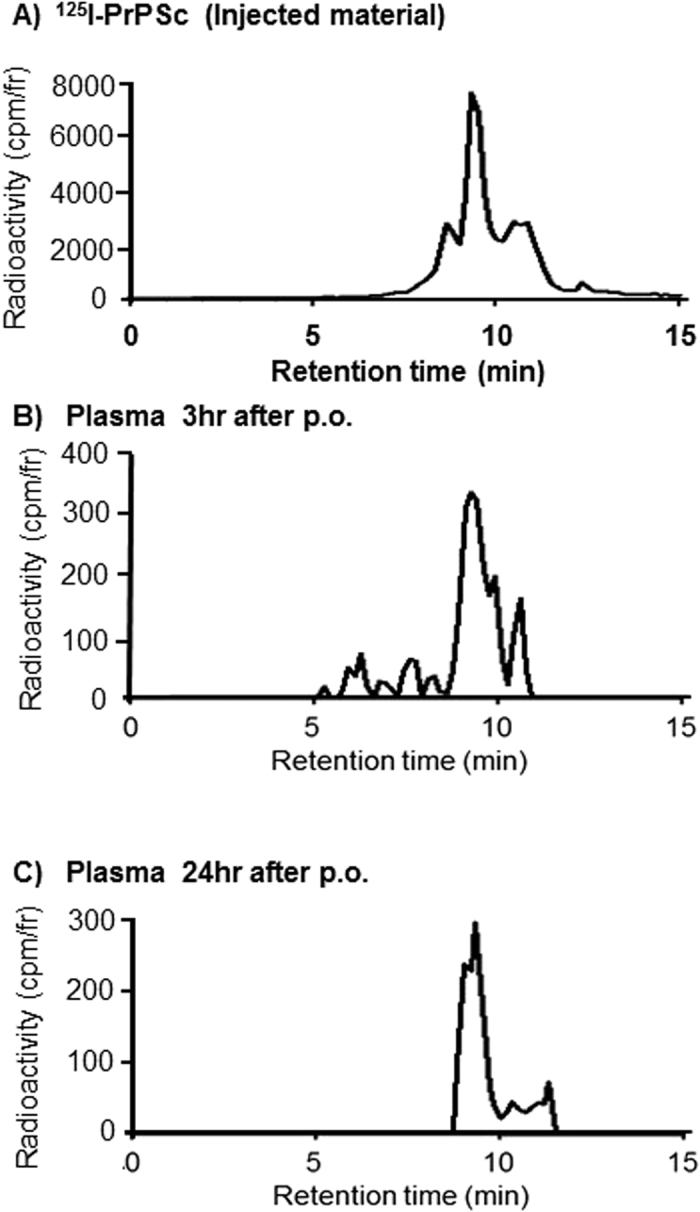
Size-exclusion radio-HPLC chromatograms of ^125^I-PrP^Sc^ in blood after p.o. administration. (**A**) Input ^125^I-PrP^Sc^. (**B**) Plasma at 3 hr after p.o. administration. (**C**) Plasma at 24 hr after p.o. administration. Plasma samples were obtained at designated times after p.o. administration of ^125^I-PrP^Sc^ in fasted mice. Radioactivity was normalized by TCA precipitation. The retention times of peaks corresponded to multimeric PrP^Sc^ as seen in the input ^125^I-PrP^Sc^. Radio-HPLC chromatograms were conducted with BioSep-Sec-S4000 column with a guard cartridge, at the flow rate of 1 ml/min of 25 mM sodium phosphate buffer, pH 7.4. Column eluates were collected at 1-min interval for first 5 min, thereafter at 10-sec per fraction. Column recovery of loaded radioactivity was >80%. Radioactivity was measured by gamma counter.

To determine whether PrP^Sc^ appearing in blood after p.o. administration contains the intact protein we performed size-exclusion chromatography. Representative radio-HPLC chromatograms including pre-administered ^125^I-PrP^Sc^ (input control), and plasma at 3 and 24 hr after p.o. administration of ^125^I-PrP^Sc^ are shown in Fig. 5. The result show that the chromatograms of the plasma samples 3 and 24 hr after p.o. administration were similar to the input ^125^I-PrP^Sc^ and largely composed of PrP^Sc^ oligomers ranging about 450-310 kDa (Fig. 5). This result indicate that oligomeric forms of ^125^I-PrP^Sc^ were effectively transported across the gut lumen and a fractional portion (0.1%ID/ml, Fig. 2A) of the agent was circulating in plasma even at long times after the administration.

### PMCA detection of PrP^Sc^ after p.o. administration

**Figure 6:**
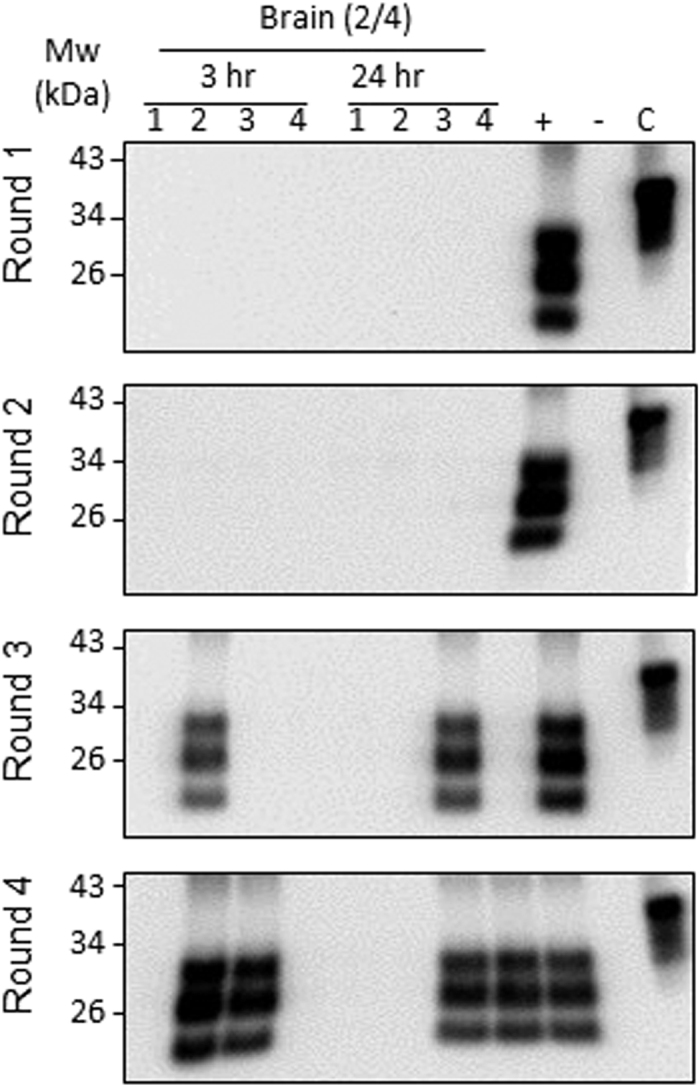
Replication-competent PrP^Sc^ in brain after p.o. administration. Whole brains from mice that received a p.o. administration of ^127^I-PrP^Sc^ were collected 3 and 24 hr post administration. Control tissue samples were from mice which received a mock injection. After precipitation with sarkosyl to remove tissue components that interfere with PMCA, samples were subjected to 4 serial rounds of PMCA, as described in Methods. Western blotting was performed with 6D11 antibody at a dilution of 1:10,000 after treatment of the samples with proteinase K. We employed non-radioactive iodine-127 to label PrP^Sc^ for PMCA analysis. Four brain samples obtained at a designated time are indicated 1 through 4, lanes + or − represent positive or negative control for PrP^Sc^ (RML), respectively. Lane C indicates non-PK digested 10% normal brain homogenate. The results indicate that 50% of brain samples have replication-competent PrP^Sc^ 3 hr and 24 hr after p.o. administration.

To study whether PrP^Sc^ absorbed in the GI tract, and distributed in peripheral organs through the blood stream retained the ability for self-replication, we subjected samples to Protein Misfolding Cyclic amplification (PMCA), a technique that mimics prion replication *in vitro*. For these studies we employed the non-radioactive isotope iodine-127 which was incorporated into PrP^Sc^ as a control for the iodination procedure. After 3 hr following p.o. administration of ^127^I-PrP^Sc^, the agent was detected in all GI-tract tissues including stomach, duodenum, jejunum, ileum, and colon by the 2^nd^ round of PMCA (Table 2 and Fig. S2). Among non-GI peripheral organs, ^127^I-PrP^Sc^ was detected in the liver as early as by the 2^nd^ round of PMCA, suggesting a first pass effect for ^127^I-PrP^Sc^ after crossing the GI tract. Lower levels of PrP^Sc^ were detected in the spleen, kidney, whole blood, and plasma 3 hr after p.o. administration. In the GI tissues and peripheral organs obtained at 24 hr after p.o. administration, PMCA amplifiable PrP^Sc^ was constantly found in these samples even though there was no detectable PrP^Sc^ in the blood and plasma 24 hr after p.o. administration, suggesting minute amounts of PrP^Sc^ deposition in these organs. PMCA with brain tissues obtained at 3 and 24 hr after p.o. administration found that brain retained self-replicating PrP^Sc^ (Fig. 6), suggesting hematogenous route of brain distribution upon oral ingestion. There was no PrP signal in tissue samples from control mice receiving a mock injection. These data suggest that PrP^Sc^ distributed in the brain and peripheral organs was not only intact but retained its capacity to sustain prion replication which is essential for the infectious property of PrP^Sc^.

## Discussion

General tissue disposition of an exogenous substance is governed by a homeostatic balance between tissue uptake and clearance. Therefore, investigating systemic pharmacokinetics and biodistribution as a dynamic system integrating the CNS and the periphery is critical to understand the consequence of body exposure to infectious prions. In the present study, we found that the absorption, distribution and accumulation of PrP^Sc^ in various organs and tissues are highly dynamic and change substantially with time after infection in relation to the routes of prion exposure. Our experimental approach does not permit to study protease-sensitive PrP^Sc^, because for radiolabeling PrP^Sc^ needs to be highly purified which necessitates strong PK treatment to remove the bulk of protein contaminants. Thus, in our study we focus exclusively on the bioavailability of the protease-resistant fraction of PrP^Sc^. While it has been reported that a large proportion of RML infectivity is associated to protease-sensitive PrP^Sc ^24, it remains to be study whether these species behave similarly to the protease-resistant forms in terms of bioavailability and pharmacokinetic properties. In this sense, it is important to highlight that in our previous study we compared the pharmacokinetic profiles of non-PK digested PrP^Sc^ (studied by western blots) and protease-resistant PrP^Sc^ (studied by radiolabeling) and both preparations showed an indistinguishable initial-phase distribution in blood^20^. In the present study, the amount of ^125^I-PrP^Sc^ orally administered was about 15.4 ng per mouse. To put this number in perspective, we compared to the quantity used for infectivity bioassays. The PrP^Sc^ concentration in a clinically sick animal has been estimated to be 0.6–2 × 10^−5^ g per g of brain^25^,^26^. For simplicity we will consider 10 μg/g of brain, in other words, 1 μg of PrP^Sc^ per ml of a 10% brain homogenate. For infection by intracerebral injection, researchers traditionally use 1–10 μl of this solution, i.e. 1–10 ng of PrP^Sc^. For oral administration, 10–100 μl of this material is commonly used, i.e 10–100 ng of PrP^Sc^. Therefore, the quantity of PrP^Sc^ used in our experiments is very similar to that used for infectivity bioassay.

The GI tract is a complex system which serves as both barrier and interface for orally ingested materials. In the stomach, only small molecules soluble in acidic conditions (pH 3), can diffuse through the gastric mucosa and epithelium. PrP^Sc^ showed bi-phasic decrease in the stomach with half-times of 1.29 hr and 25.9 hr. The intestinal epithelium has close contact with the agents being further digested which can be transformed and taken in the villi through local mucosal layers. It has been shown that transcellular uptake of infectious prion particles was mediated through normal enterocytes and Peyer’s patches via M-cells^11^. In prior studies with artificial spherical materials suggested that physico-chemical characteristics of the particles, such as physical size^27^ the surface charge^28^, and surface ligands, affect the translocation behavior of ingested agents in the GI tract, including the Peyer’s patches^29^. In the present study, size exclusion HPLC analysis found that plasma contained oligomeric PrP^Sc^ indicating orally administered PrP^Sc^ diffused through intestinal mucosal layers, crossed epithelial cell layers, and absorbed into serosal compartment in the GI tract. The molecular weights of the PrP^Sc^ oligomers used in this study and detectable in blood after p.o. administration were in the range of 310–450 KDa. Even though the most infectious PrP^Sc^ particles have been reported to be in this range for hamster 263 K prions^21^, as well as various ovine prion strains^30^, it is unclear that the situation will be the same for the RML prion strains. Importantly, PrP^Sc^ retrieved from blood after p.o. administration retained the capacity to induce the conversion of PrP^C^ in PMCA experiments, suggesting the material is competent to act as an infectious agent. This view is also supported by a recent study reporting that seeding-competent PrP^Sc^ appeared in blood soon after p.o. administration of infected materials in hamsters and cervids^31^.

In our previous studies^20^,^22^,^23^, we reported that purified PrP^Sc^ injected i.v. can reach the brain exclusively by crossing the blood-brain barrier, without the involvement of peripheral nerves, and PrP^Sc^ appeared in both the brain parenchyma and the cerebrospinal fluid after i.v. administration^22^. In the current study, we found that the brain received ~0.03%ID of PrP^Sc^ per g of brain after p.o. administration (Fig. 4A) which represents ~4.5 pg of PrP^Sc^ per g of brain weight. Since blood contains 1.1%ID/ml of PrP^Sc^, it is possible to estimate that ~3.3 pg of PrP^Sc^ are present in the cerebral blood vessel per g of brain, based on the brain vascular space^20^. Thus, brain parenchymal distribution could be up to ~1.2 pg per g of brain. As stated before, we have estimated that the PrP^Sc^ concentration in the brain of a terminally sick animal is around 0.6–2 × 10^−5^ g per g of brain^25^,^26^. Since in these models the last infectious dilution of brain homogenate injected by intra-cerebral inoculation is ~10^−8 ^32, we estimate that the minimum quantity of PrP^Sc^ that can initiate disease in the brain is ~0.2 pg per g of brain. Thus, numerically, the amount of PrP^Sc^ that reached the brain from the hematogenous route after oral administration appears to be comparable to the minimum amount needed to initiate the disease. We are aware that this view may seem contradictory to previous studies^33^,^34^,^35^,^36^ which have solidly established that neuroinvasion is dependent of peripheral replication of PrP^Sc^ in lymphatic tissues including secondary lymphatic organs and transport thru the peripheral nerves. Our present findings do not intend to contradict these widely accepted pathways for neuroinvasion, but merely make the point that the amount of prions reaching the brain directly by the hematogenous route is not negligible and the exact contribution of this route should be further investigated.

Also, our findings do not diminish the importance of the lymphatic system and retrograde nerve transport of prions into the CNS. On the contrary, our data showing high affinity and rapid association of orally administrated PrP^Sc^ to PBMC further confirms the involvement of the lymph-reticular system in prion diseases. Indeed, after oral administration up to 87% of PrP^Sc^ in blood was cell-associated whereas only ~20% was present in blood cells when the protein was directly loaded into the blood. Considering the volume of buffy coat fraction in whole blood is <1%, the fact that PBMCs contain 17% of ^125^I-PrP^Sc^ present in blood (Fig. 3A), suggests a higher affinity of PBMCs for PrP^Sc^ compared to RBCs. We estimate that PBMCs contain about 1000 times higher concentrations of PrP^Sc^ than RBCs when the number of cells in whole blood is taken into account (Fig. 3C). Nevertheless, the large amount of PrP^Sc^ in the RBCs fraction (48%) suggests these cells may serve as a reservoir of PrP^Sc^ after p.o. administration. The blood/plasma ratio indicates that significant amount of PrP^Sc^ present in blood at long times after p.o. administration (>12 hr) is cell-associated, suggesting a delay in biological clearance of this portion of PrP^Sc^. Surprisingly, PrP^Sc^ directly injected into blood stream largely remained in plasma fraction, and only about 20% of PrP^Sc^ got cell associated (Fig. 3B). The differences in blood cell association of PrP^Sc^ can be explained by the fact that PrP^Sc^ crossing the GI tract is immediately exposed to lymphatic tissues and follicle associated white blood cells^13^. On the contrary, PrP^Sc^ directly infused into vascular system remains in plasma fraction after saturating bindings to circulating blood cells. Considering that different routes of exposure to prions lead to substantial differences in PrP^Sc^ distribution in distinct blood fractions, it is likely that whole body distribution of prions may also differ at different stages of the disease when prions could be shed into blood from either peripheral replication or leakage from the brain^25^.

By comparing pharmacokinetic parameters obtained through p.o. and i.v. administration (Table 1), we estimated the percentage of PrP^Sc^ that was absorbed into the body. The estimated bioavailability of prions was 33.6%, which is very high considering the protein nature of the infectious agent. PrP^Sc^ crosses the intestinal barrier as an oligomeric form that retains the capacity to self-replicate. Once PrP^Sc^ became blood-borne, its distribution half-life was about 5 min, tissue sequestration occurred in the brain and majority of peripheral organs, without much degradation of PrP^Sc^ in blood^20^. A caveat from our study is that the pharmacokinetic parameters obtained in mice cannot directly translate to humans. In addition, the use of stomach gavages instead of putting the material into the animal food may not replicate exactly the natural situation. The reason we used stomach gavage administration because this procedure gives the best control over the amount that is really getting in the gastro-intestinal system. The exposure route and amount of infectious prions upon initial contact in natural case may be less robust compared to present experimental setting. Nevertheless, our data show that intact, replication-competent PrP^Sc^ can effectively cross the intestinal barrier, distribute in blood flow, and temporally appeared in the brain.

## Methods

### Ethics statement

All animal experiments were approved by and conducted in strict accordance with guidelines of the Animal Care and Use Committee of the University of Texas Health Science Center in Houston and complied with the recommendations in the Guide for the Care and Use of Laboratory Animals of the National Institutes of Health and the Reporting of *in Vivo* Experiments Guidelines.

### Purification of PrP^Sc^ from infected mouse brain

PrP^Sc^ was purified from mice infected with Rocky Mountain Laboratory (RML) scrapie strain as previously described^20^,^37^,^38^. Briefly, brain tissue was homogenized at 10% w/v in phosphate buffered saline containing 10% sarkosyl and subjected to a series of differential centrifugations employing a Beckman TL-100 ultracentrifuge (OptimaMAX Ultracentrifuge, Beckman-Coulter) with the final step consisting of a sucrose gradient. The material was then treated with proteinase K (PK) (100 μg/ml) at 37 °C for 2 hr followed by ultracentrifugation to precipitate PrP^Sc^. The purity of PrP^Sc^ was confirmed by silver staining and estimated to be >95%. PrP^Sc^ concentration was measured by micro BCA protein assay reagent (Pierce).

### Radiolabeling

Purified PrP^Sc^ and albumin (Fraction G, bovine serum albumin, Sigma) were radioactively labeled by the iodobead method with [^125^I]Na (Perkin-Elmer) or [^127^I]Na (Sigma), as previously described^20^,^39^. Briefly, ultra-purified PrP^Sc^ (10 μg) or albumin (5 μg) was mixed with [^125^I]Na (2 mCi) or equivalent amount of ^127^INa in 250 mM chloride-free sodium phosphate buffer (pH 7.4), and the protein labeling was initiated by adding one iodobead into the mixture. After 15 min, the reaction was terminated by removing the bead from the mixture. Each labeled agent was purified by Sephadex G-10 chromatography to remove free iodine. The labeled PrP^Sc^ or albumin was diluted with phosphate buffered saline and further centrifuged in albumin pre-coated Microcon filtration tube (Mw cutoff: 10 kDa) at 12,000 rpm for 30 min to further remove free iodine from the G-10 eluate. Labeled PrP^Sc^ and albumin was extensively washed by this procedure. ^125^I-PrP^Sc^ had a specific radioactivity of 1.3 × 10^5^ cpm/ng. The radioactively labeled PrP^Sc^ preparations we used had over 95% precipitation with trichloroacetic acid (TCA). Materials were freshly prepared on the day of the experiment.

### Animals Procedures

Female C57BL/6 mice (Charles River) were studied at 10 to 12 weeks of age (25.1 ± 1.2 g of body weight). Mice anesthetized with Avertin (2%) received a per oral (p.o.) or intravenous (i.v.) administration of ^125^I-PrP^Sc^ (2 × 10^6^ cpm for pharmacokinetic study, 1 × 10^6^ to 2 × 10^7^ cpm for radio-HPLC and ficoll fractionation studies), ^127^I-PrP^Sc^ (15 ng, equivalent to 2 × 10^6^ cpm of ^125^I-PrP^Sc^), or ^125^I-albumin (5 × 10^5^ cpm). At 0.17 (10 min), 0.5 (30 min), 1, 3, 6, 12, 18, and 24 hr after administration, mice were sacrificed, and the blood, brain, liver, spleen, kidney, stomach, duodenum, jejunum, and colon were immediately collected and weighed. Plasma from mouse whole blood was isolated by centrifugation. Radioactivity was measured by a gamma counter (COBRA II, Packard). TCA precipitation of radioactively labeled PrP^Sc^ in each sample was also measured after administration and results normalized by the precipitation ratio (%).

### Separation of blood fractions by Ficoll-Paque density gradient

Whole blood was diluted with 2-fold volume of phosphate buffered saline, and gently floated onto equal volume of Ficoll-Paque Premium (GE Healthcare, London). The sample was centrifuged at 400 × g for 30 minutes at room temperature. Fractions were separated into plasma, PBMC, and RBC fractions. The radioactivity from ^125^I-PrP^Sc^ was measured after TCA precipitation of each fraction by a gamma counter (Packard), and results were presented as %ID/ml of original whole blood volume. Representative numbers of cells in PBMC and RBC fractions were counted, and the values were 1 × 10^6^ cells/ml and 4 × 10^8^ cells/ml of whole blood, respectively.

### Pharmacokinetic analysis

The arithmetic mean values of concentration-time profiles of intact ^125^I-PrP^Sc^ in whole blood and plasma were used for pharmacokinetic analysis based on the statistical moment theory according to Yamaoka and colleagues^40^, using the following equations:


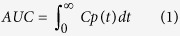



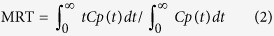


where, AUC represents the area under the concentration-time curve, MRT indicates the mean residence time in the body, Cp(t) is defined as the concentration of ^125^I-PrP^Sc^ at time t. AUC_(0–∞)_ and MRT were estimated by linear trapezoidal integration with extrapolation to infinite time. The total body clearance (CL_tot_) and steady-state volumes of distribution (Vd_ss_) were calculated by CL_tot_ = injected dose (ID)/AUC_(0–∞)_, and Vd_ss_ = MRT × CL_tot_, respectively. Terminal phase rate constant (k) was determined by least-square regression analysis of terminal log-linear portion of the concentration time curve of whole blood and plasma, and terminal half-life (t_1/2_) was calculated as ln2/k. T_max_ indicates the time to reach the maximal concentration (C_max_). These were determined from the concentration time profile of ^125^I-PrP^Sc^ in whole blood and plasma. Bioavailability (BA) was calculated by the following equation:


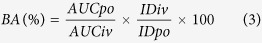


where AUC and ID accompanied with a suffix represent the value obtained by each injection route.

To estimate gastric emptying half-time, the concentration-time profile for ^125^I-PrP^Sc^ in the stomach was fitted with following bi-exponential equation using the nonlinear least squares method,


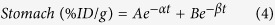


where A and B represent y-intercepts at the initial and late phases after p.o. administration, respectively. The rate constants in initial (*α*) and late (β) phases were employed to estimate gastric emptying half-times as ln2/*α* and ln2/β, respectively.

### HPLC analysis

The stability of ^125^I-PrP^Sc^ in the plasma, blood, brain, liver, spleen, kidney, stomach, duodenum, jejunum, and colon was examined using HPLC. Mice received a p.o. administration of ^125^I-PrP^Sc^ of 1 × 10^6^ or 2 × 10^7^ cpm/mouse. The samples from animals sacrificed at 3, or 24 hr were used for the HPLC analysis. Plasma (300 μl) was directly loaded onto the HPLC column. The tissues were mechanically homogenized in a 9-fold volume of mobile phase, centrifuged at 20,000 × g for 20 minutes, and the supernatant was collected. Each tissue extract was injected onto HPLC (Shimadzu UPLC system LC-20AB). Radio-HPLC chromatography was conducted with the size exclusion column BioSep-SEC-S4000 (7.8 mm × 300 mm, Phenomenex, CA) and a guard cartridge. The mobile phase consisted of 25 mM sodium phosphate buffer (pH 7.4). Fractions were collected at 1-min interval for first 5 min, thereafter at 10-sec per fraction at the flow rate of 1.0 ml/min. Column recovery of loaded radioactivity was >80%. Radioactivity was measured by gamma counter. TCA precipitation of fractions was also performed. The retention time for molecular size markers was separately measured, and the markers included thyroglobulin (669 kDa), aldolase (158 kDa), conalbumin (75 kDa), carbonic anhydrase (29 kDa), aprotinin (6.5 kDa), and iodine-125 (125 Da). UV absorbance at 280 nm was used to characterize the retention time of the markers, except for iodine-125 which was characterized by radioactivity. All markers were obtained through commercial sources.

### PMCA procedure

Aliquots of purified PrP^Sc^ either iodinated or not were used to seed conversion of mouse PrP^C^ in 10% normal mouse brain homogenate. The detailed protocol for PMCA has been described elsewhere^41^,^42^. Briefly, tissues from infected animals were homogenized (10% w/v) in phosphate buffered saline containing complete protease inhibitors (Roche) by a mechanic homogenizer and hard tissue tubes. Tissue homogenates were incubated with equal volume of 20% sarkosyl solution for 10 min in agitation at room temperature. Thereafter, the mixture was centrifuged at high speed (100,000 × g) for 60 min at 4 °C. Pellet was washed with one volume of PBS and centrifuged for 30 mins at the same speed. The final pellet was resuspended in 100 μL of 10% normal brain homogenate for PMCA reaction. Four rounds of PMCA were conducted, and durations of PMCA reaction per round were 3 days for 1^st^ round and 2 days for 2^nd^ through 4^th^ round. Reaction tubes were positioned on an adaptor placed on the plate holder of a microsonicator (Misonix Model Q700) and programmed to perform sonication cycles of 20 sec every 30 min at an amplitude of 20, in an incubator set at 34 °C.

### SDS-PAGE and Western Blotting

Samples were boiled in NuPage sample buffer (Invitrogen) supplemented with 300 mM DTT, analyzed by SDS-PAGE and electroblotted into 0.45 μm nitrocellulose membranes (Amersham). PrP was probed by 6D11 antibody (Covance, dilution 1:10,000) and anti-mouse secondary antibody (sigma). The immunoreactive bands were visualized by enhanced chemiluminescence assay (ECL prime, Amersham), and the signal was processed by a digital imaging analyzer (Bio-Rad dark room imaging system).

### Statistical analysis

Means are presented with their standard errors and compared by one-way analysis of variance (ANOVA) followed by Newman-Keuls multiple comparison test, two-way ANOVA followed by the Bonferroni’s test, or by two-tailed unpaired t-test with Welch’s correction. Statistical analysis was done with the Prism 5.0 program (GraphPad).

## Additional Information

**How to cite this article**: Urayama, A. *et al*. Prions efficiently cross the intestinal barrier after oral administration: Study of the bioavailability, and cellular and tissue distribution *in vivo*. *Sci. Rep.* **6**, 32338; doi: 10.1038/srep32338 (2016).

## Supplementary Material

Supplementary Information

## Figures and Tables

**Table 1:** Pharmacokinetic parameters for ^125^I-PrP^Sc^.

	P.O. administration		I.V. injection	
	Whole Blood	Plasma	Whole Blood	Plasma
Tmax (hr)	3.0	0.5	—	—
Cmax (%ID/ml)	1.1	0.69	—	—
Terminal t_1/2_ (hr)	15.7	6.45	6.05	5.53
MRT (hr)	20.7	8.64	6.04	5.74
CLtot (ml/hr)	1.87	1.89	2.21	1.88
Vdss (ml)	38.8	16.3	13.4	10.8
AUC_0-∞_ (%ID/ml × hr)	17.9	7.59	45.9	53.2
Bioavailability, F (%)	33.6	14.3	—	—

Mean values are reported. Pharmacokinetic analyses were based on the time-courses of ^125^I-PrP^Sc^ shown in Fig. 2. Tmax: time that maximal concentration was observed. Cmax: maximal concentration at Tmax. Terminal t_1/2_: half-life of ^125^I-PrP^Sc^, MRT: mean residual time, CLtot: total body clearance, Vdss: volumes of distribution, AUC: area under the time curve, and ID: injected dose.

**Table 2:** Detection of PrP^Sc^ by protein-misfolding cyclic amplification in biological samples after p.o. administration in fasted mice.

PMCA Round	3 hr after P.O.				24 hr after P.O.			
	1	2	3	4	1	2	3	4
Stomach	**4/4** (100%)	**4/4** (100%)	**4/4** (100%)	**4/4** (100%)	**2/4** (50%)	**3/4** (75%)	**4/4** (100%)	**4/4** (100%)
Duodenum	**2/4** (50%)	**4/4** (100%)	**4/4** (100%)	**4/4** (100%)	**1/4** (25%)	**2/4** (50%)	**3/4** (75%)	**3/4** (75%)
Jejunum	**1/4** (25%)	**3/4** (75%)	**4/4** (100%)	**4/4** (100%)	**2/4** (50%)	**3/4** (75%)	**4/4** (100%)	**4/4** (100%)
Ileum	**1/4** (25%)	**4/4** (100%)	**4/4** (100%)	**4/4** (100%)	**2/4** (50%)	**3/4** (75%)	**4/4** (100%)	**4/4** (100%)
Colon	**0/4** (0%)	**3/4** (75%)	**3/4** (75%)	**3/4** (75%)	**0/4** (0%)	**2/4** (50%)	**4/4** (100%)	**4/4** (100%)
Spleen	**0/4** (0%)	**1/4** (25%)	**2/4** (50%)	**3/4** (75%)	**0/4** (0%)	**0/4** (0%)	**1/4** (25%)	**1/4** (25%)
Liver	**0/4** (0%)	**4/4** (100%)	**4/4** (100%)	**4/4** (100%)	**1/4** (25%)	**1/4** (25%)	**2/4** (50%)	**3/4** (75%)
Kidney	**0/4** (0%)	**0/4** (0%)	**3/4** (75%)	**4/4** (100%)	**0/4** (0%)	**0/4** (0%)	**0/4** (0%)	**1/4** (25%)
Whole blood	**0/4** (0%)	**1/4** (25%)	**1/4** (25%)	**1/4** (25%)	**0/4** (0%)	**0/4** (0%)	**0/4** (0%)	**0/4** (0%)
Plasma	**0/4** (0%)	**0/4** (0%)	**0/4** (0%)	**1/4** (25%)	**0/4** (0%)	**0/4** (0%)	**0/4** (0%)	**0/4** (0%)

Protein-misfolding cyclic amplification (PMCA) was performed with biological samples obtained at 3 and 24 hr after p.o. administration of ^127^I-PrP^Sc^ in fasted (16 hr) mice. Four separate sets of samples were tested and values were presented as the number of PMCA positive samples over four determinations. The percentage of PMCA positive samples were also reported in parenthesis. Representative blotting results of PMCA reaction are shown in Fig. S2.
