# Supplementary material for: Prions efficiently cross the intestinal barrier after oral administration: Study of the bioavailability, and cellular and tissue distribution in vivo
Source: Sci Rep. 2016 Aug 30;6:32338. doi: 10.1038/srep32338 (PMC5004172; doi:10.1038/srep32338)
Supplement: Supplementary Information [file srep32338-s1.pdf]

[Supplementary Information]

Prions efficiently cross the intestinal barrier after oral administration: Study of the bioavailability, and cellular and tissue distribution *in vivo*

Akihiko Urayama[1]*, Luis Concha-Marambio[1,2], Uffaf Khan[1], Javiera Bravo-Alegria[1,2], Vineetkumar Kharat[1], and Claudio Soto[1,2]*

[1] Mitchell Center for Alzheimer's Disease and Related Brain Disorders, Department of Neurology, University of Texas Medical School at Houston, Texas, United States of America
[2] Universidad de los Andes, Santiago, Chile.

Fig. S1

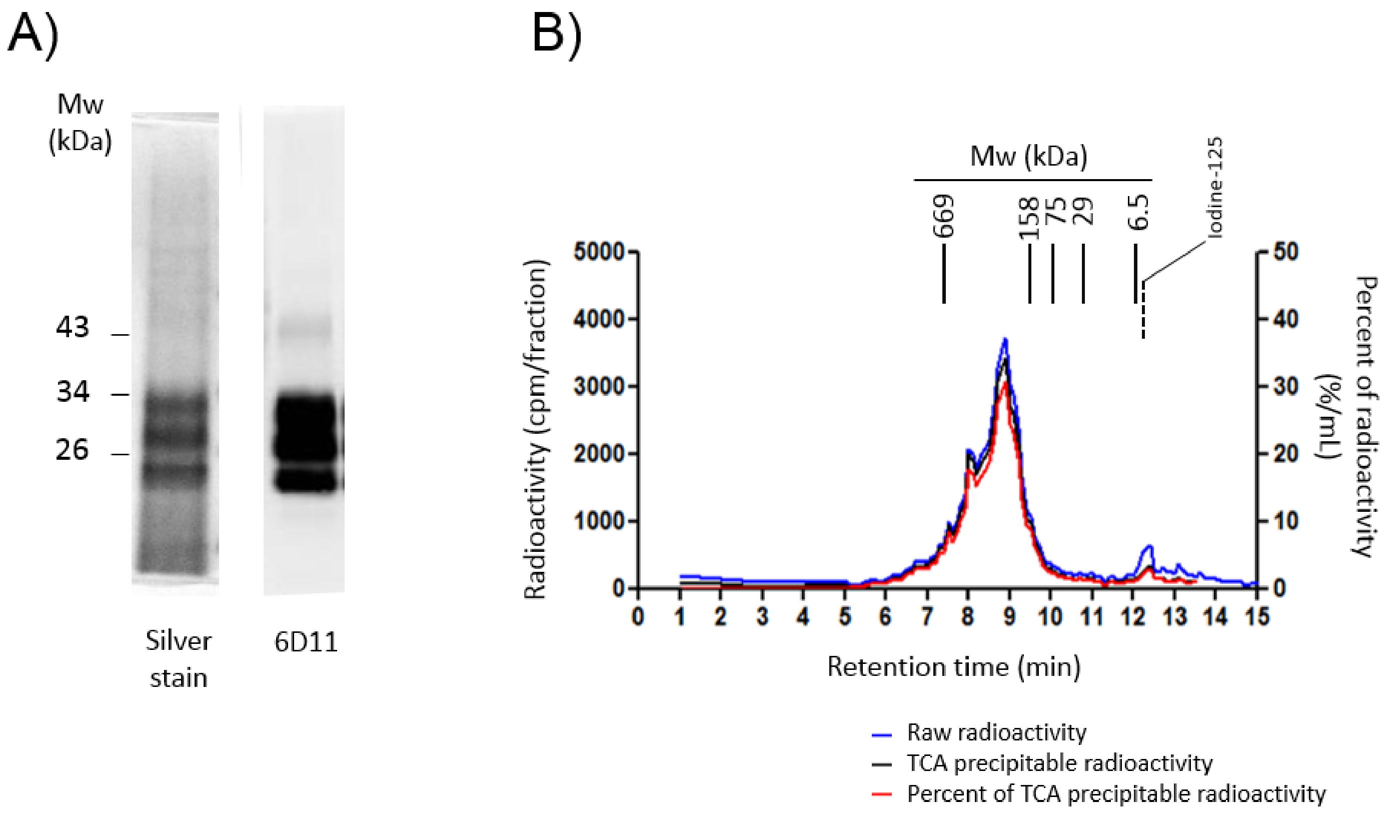

Figure S1. Purification of PrP^{Sc} from infected mouse brain and radiometric characterization of ^{125}I-PrP^{Sc} by size exclusion chromatography.

(A) Silver staining and western blotting of purified PrP^{Sc} probed by 6D11 anti-PrP antibody. Clear PrP^{Sc} bands were observed as well as some aggregation forms in both Western blotting and silver stain. There was no reactivity associated with PrP^{Sc} in lower-molecular-mass fractions. **(B)** Size-exclusion radio-HPLC chromatogram of radiolabeled ^{125}I-PrP^{Sc}. Radiolabeled PrP^{Sc} was eluted as multimeric forms raging the retention times from 6 to 11 min through a size-exclusion column. Raw radioactive count, TCA precipitable count (left axis), and the percent of radioactivity loaded onto the column (right axis) are plotted, respectively. Molecular weight markers were also plotted with the retention time for each marker.

Fig. S2

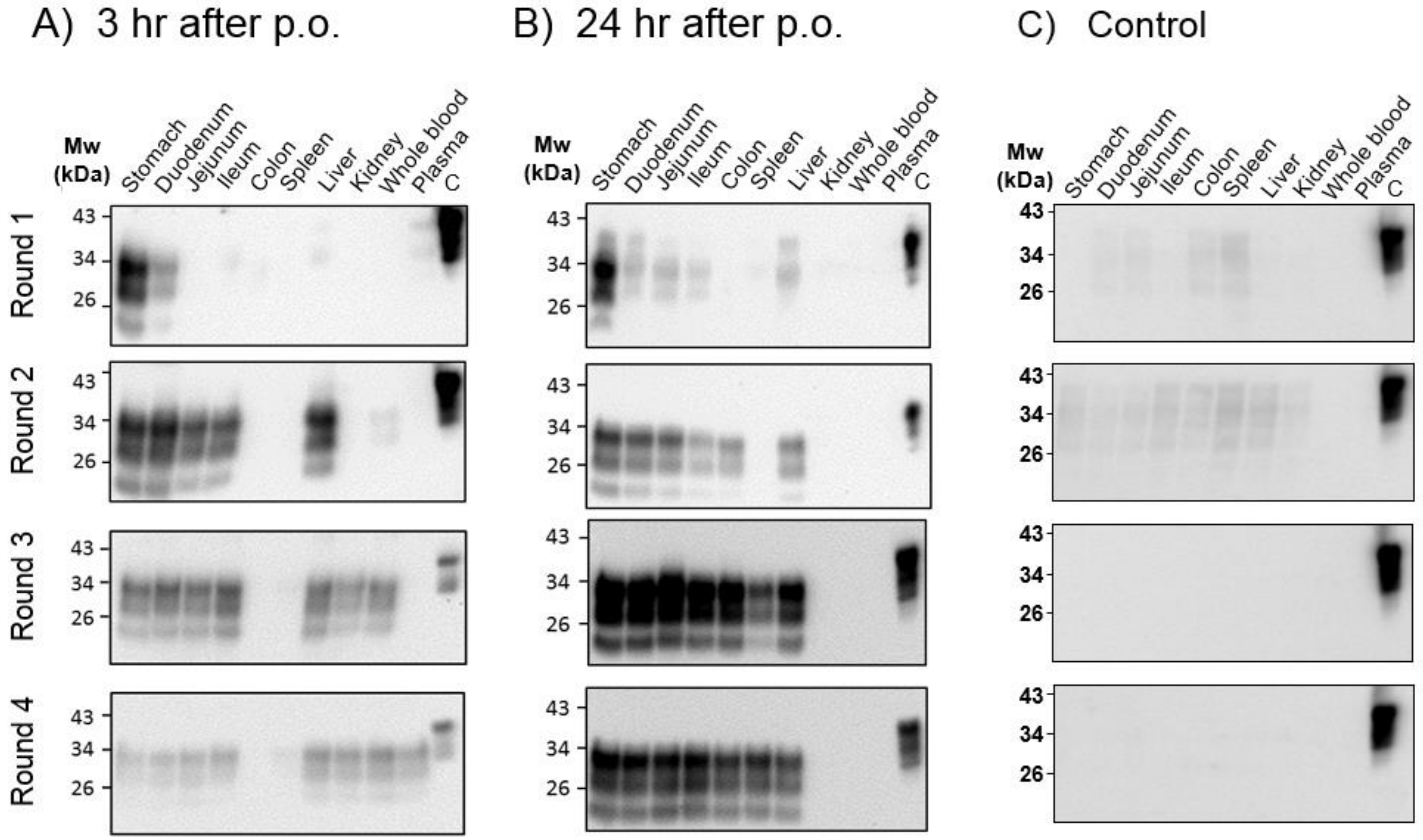

Figure S2. Detection of replication competent PrP^{Sc} by PMCA in different tissues after p.o. administration in fasted mice.

(A) PMCA amplifications of PrP^{Sc} with samples obtained at 3 hr after p.o. administration of ^{127}I-PrP^{Sc}. **(B)** PMCA amplifications of PrP^{Sc} with samples obtained at 24 hr after p.o. administration of ^{127}I-PrP^{Sc}. **(C)** Control tissue samples from mice that received a mock injection. Representative PMCA results are presented, and whole data are summarized in Table 2. Tissue extracts and fluid samples were subjected to 4 serial rounds of PMCA, as described in Methods. Western blotting was performed with 6D11 antibody at a dilution of 1:10,000 after treatment of the samples with proteinase K. We employed non-radioactive iodine-127 to label PrP^{Sc} for PMCA analysis. Lane C in each panel indicates non-PK digested 10% normal brain homogenate.
